# Immunocytokines with activity-on-demand by combination with small molecule inhibitors

**DOI:** 10.1038/s44321-024-00047-9

**Published:** 2024-03-06

**Authors:** Dafne Müller

**Affiliations:** Institute of Cell Biology and Immunology, University of Stuttgart, Allmandring 31, 70569 Stuttgart, Germany

**Keywords:** Cancer, Immunology, Pharmacology & Drug Discovery

## Abstract

D. Mueller discusses the study by Rotta et al, in this issue of *EMBO Mol. Med.*, that presents a strategy to control adverse events related to tumor-targeted cytokine therapy without interfering with its therapeutic efficacy by combining tumor-targeted IL-12 with a JAK inhibitor.

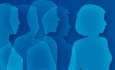

## Teaser

Dose-limiting systemic toxicity constitutes a major impediment to the application of cytokines in cancer therapy. To enhance the therapeutic index, tumor-directed antibody-cytokine fusion proteins, i.e., immunocytokines, are developed for targeting-mediated cytokine enrichment at the tumor site, allowing for an effective local concentration at a lower dosage. However, the therapeutic window is narrow, making strategy improvements to further reduce off-target toxicity of great interest. Recently, the combination with a small molecule inhibitor of the cytokine signaling pathway has been proposed to suppress systemic toxicity during the delivery stage of the immunocytokine without interfering with its therapeutic efficacy. In this issue of *EMBO Molecular Medicine*, proof of concept is provided by Rotta et al in preclinical studies on tumor-targeted IL-12 in combination with a JAK inhibitor.

Immunocytokines, comprising various antibody formats and cytokines, have been reported for cancer therapy (Gout et al, 2022). Targeting efficacy was shown to be determined not only by the antibody but also significantly influenced by the particular cytokine, with potential implications for off-target toxicity (Hutmacher and Neri, 2019). Most advanced in clinical trials are immunocytokines with the cytokines IL-2, TNF, and IL-12 (Rybchenko et al, 2023). These pro-inflammatory cytokines are vasoactive and can induce further cytokine production, potentially causing adverse events such as hypotension, nausea, vomiting, flu-like symptoms, and, in some cases liver function abnormalities (Rudman et al, 2011; Johannsen et al, 2010). To enhance tolerability, slow infusion or repeated administration of low doses for progressive cytokine enrichment at the tumor site is proposed, as well as intralesional administration (Hutmacher and Neri, 2019; Neri, 2019). Current preclinical developments also include selective or activity-reduced mutants and on-site assembling of sequentially delivered subunits of the cytokine payload (Gout et al, 2022). Furthermore, the combination with small-molecule inhibitors temporarily blocking the cytokine signaling pathway started to be explored (Dakhel et al, 2019; Rotta et al, 2024). This is in line with an emerging interest in using small-molecule inhibitors to support other targeted immunotherapeutic strategies. In the context of T cell-engaging therapies, FDA-approved tyrosine kinase inhibitors, targeting pathways downstream of TCR activation and cytokine receptors, are being investigated to mitigate the cytokine release syndrome (CRS) (Leclercq et al, 2022). T-cell engagers are bispecific antibodies that induce tumor cell killing by redirecting T-cell cytotoxicity toward tumor cells. However, on-target T-cell activation can also initiate the release of pro-inflammatory cytokines, potentially leading to a cytokine storm with severe clinical implications. Combination with tyrosine kinase inhibitors, preventing T cell-derived cytokine release, is expected to suppress these side effects. However, the balance in reducing the risk of CRS without compromising therapeutic efficacy is challenging for leukemia-directed T-cell engagers. In preclinical studies, reversible off-switch capacity of cytokine release and cytotoxicity was reported for a dual Bcr-Abl/Scr inhibitor. Interestingly, selectivity was observed for mTOR and JAK inhibitors, suppressing cytokine release while retaining bispecific antibody-mediated anti-tumor activity (Leclercq-Cohen et al, 2023). Adequate translation into clinical settings remain to be investigated. In the context of immunocytokines, the concept of the combinatory approach is focused on solid tumors. Administration of a pathway-selective small-molecule inhibitor suppresses the cytokine activity temporarily during the systemic delivery of the immunocytokine without compromising later the activity of the immunocytokine retained in the tumor. Thus, taking advantage of the short PK/PD of the inhibitor and tumor targeting-mediated accumulation of the immunocytokine, on-demand-activity can be achieved at the tumor site. In preclinical studies, this principle was initially introduced by the combination of a tumor stroma-directed immunocytokine with TNF (L19-TNF) and a RIPK1 inhibitor (GSK’963) (Dakhel et al, 2019). Now, it was demonstrated for the combination of the tumor stroma-directed immunocytokine with mouse IL-12 (L19-mIL-12) and the JAK2 inhibitor ruxolitinib (Rotta et al, 2024). In dose escalation studies in a syngeneic tumor mouse model, pretreatment with ruxolitinib enhanced the tolerability of L19-mIL-12 without interfering with the anti-tumor effect. Consistently, analysis of immune cells and the tumor proteome 24 h after the last treatment indicated the persistence of the cytokine-induced immune response. Furthermore, analysis focusing on “on-target off-tumor” toxicity revealed inhibitor-mediated reduction in body weight loss and partial protection from liver toxicity. Subsequently, the authors assessed treatment adjustment to prolong the masking activity of ruxolitinib by a repeated, adequately spaced injection schedule that led to further improvement in reducing symptoms of toxicity. Thus, the potential of the combinatory strategy for regulatory fine-tuning was evidenced, showing promise for potential clinical development.

In summary, Rotta et al, 2024 provide additional proof of concept for the “Intra-Cork” strategy, i.e., the combined application of an immunocytokine with a selective small-molecule inhibitor to block cytokine activity in the periphery, confining the therapeutic activity to the tumor site. This work will contribute to facilitate the clinical development and encourage future investigations on fine-tuning the approach and extending the principle to other immunocytokines and pathway-specific signaling inhibitors. This might open novel therapeutic avenues for the safe application of immunocytokines in cancer therapy.
